# COVID-19 vaccine uptake among people who inject drugs in Tijuana Mexico

**DOI:** 10.3389/fpubh.2022.931306

**Published:** 2022-09-06

**Authors:** Alicia Harvey-Vera, Sheryl Munoz, Irina Artamonova, Daniela Abramovitz, Maria Luisa Mittal, Cecilia Rosales, Steffanie A. Strathdee, Maria Gudelia Rangel

**Affiliations:** ^1^Division of Infectious Diseases and Global Public Health, Department of Medicine, University of California, San Diego, San Diego, CA, United States; ^2^US-Mexico Border Health Commission, Tijuana, Mexico; ^3^Escuela de Medicina, Campus Tijuana, Universidad Xochicalco, Tijuana, Mexico; ^4^Mel and Enid Zuckerman College of Public Health, University of Arizona, Tucson, AZ, United States; ^5^Departmento de Estudios de Población, El Colegio de la Frontera Norte, Tijuana, Mexico

**Keywords:** COVID-19, substance use, people who inject drugs, COVID-19 vaccines, sex work

## Abstract

**Background:**

SARS-CoV-2 prevalence is elevated among people who inject drugs (PWID). In Tijuana, Mexico, COVID-19 vaccines became available to the general population in June 2021, but uptake among PWID was <10%. We studied COVID-19 vaccine uptake among PWID in Tijuana following implementation of a pop-up vaccination clinic.

**Methods:**

Beginning in October, 2020, PWID in Tijuana aged ≥18 years were enrolled into a longitudinal cohort study. At baseline and semi-annually, participants underwent interviewer-administered interviews on health behaviors and COVID-19 exposures through April 5, 2022. From June 21—September 20, 2021, staff referred PWID to a temporary COVID-19 vaccine pop-up clinic that was coincidentally established near the study office. Participants attending the clinic completed a short interview on barriers to vaccination and were offered facilitated access to free Janssen® COVID-19 vaccine. All participants were reimbursed $5 for this interview, regardless of whether or not they chose to be vaccinated. Poisson regression was used to evaluate the effect of the pop-up clinic on COVID-19 vaccination uptake, controlling forpotential confounders.

**Results:**

Of 344 participants, 136 (39.5%) reported having received at least one COVID-19 vaccine dose during the 10 months follow-up period, of whom 113 (83.1%) received vaccine at the pop-up clinic and 23 (16.9%) elsewhere. One third of those receiving COVID-19 vaccine during the pop-up clinic were previously vaccine hesitant. Attending the pop-up clinic was independently associated with higher rates of COVID-19 vaccination Adjusted Rate Ratio (AdjRR: 9.15; 95% CI: 5.68–14.74).

**Conclusions:**

We observed a significant increase in COVID-19 vaccine uptake associated with attending a temporary pop-up vaccine clinic in Tijuana suggesting that efforts to improve vaccination in this vulnerable population should include convenient locations and staff who have experience working with substance using populations. Since COVID-19 vaccination rates remain sub-optimal, sustained interventions to increase uptake are needed.

## Introduction

Individuals with a diagnosis of substance use disorder, including opioid use disorder, have significantly higher risk of acquiring SARS-CoV-2 and worse clinical outcomes than other COVID-19 patients (1). In a previous study of people who inject drugs (PWID) in Tijuana and San Diego, SARS-CoV-2 prevalence was 37.5%, which was higher than the general population in both cities (2). PWID may experience severe COVID-19 illness due to comorbid conditions, including chronic kidney, liver and lung diseases (1, 3–5). Additionally, PWID have limited access to health care services and often experience stigma and discrimination that perpetuates medical mistrust, contributing to poor health care utilization (6, 7). Due to the high COVID-19 burden among PWID, there is a need to expand COVID-19 vaccination efforts for this population (2, 8, 9).

In Mexico, SARS-CoV-2 has caused over 5.5 million cases of COVID-19 (10). Mexico developed five stages for vaccination rollout (11). In Stage 1, Mexico vaccinated 100% of their healthcare personnel (1.25 million people) between December 2020 and February 2021 (12). In Stage 2 (February to May 2021), vaccination efforts were prioritized to municipalities with concentrated COVID-19 mortality, starting with those 60 years old and older (11, 13). Efforts expanded with Stage 3 (May to June 2021) prioritizing pregnant women in the second or third trimester and persons 50 years or older. Stage 4 (June to July 2021) expanded to persons 40 years or older, and finally, Stage 5 vaccinating the general population beginning in July 2021 (12). In Tijuana, Baja California, Mexico's most northwestern state abutting California, United States, COVID-19 vaccines became available according to the above schedule at mass vaccination sites throughout the city, where SinoVac-CoronaVac®, Oxford-AstraZeneca®, and J&J/Janssen® vaccines were offered (14).

By June 25, 2021, Baja California reported being close to becoming the first state in Mexico to achieve full vaccination for most of its adult residents (15, 16) following a donation of J&J/Janssen vaccine from the United States; however, vaccine uptake was lower in some marginalized populations (17). As in other Mexican cities, individuals in Tijuana seeking COVID-19 vaccination were required to register online and enter their CURP (*Clave Única de Registro de Población*; Mexican official unique identifier) to obtain an appointment and were asked to print the appointment card or present their CURP to verify their identity (18), which has been shown to be a barrier to health services in other settings (19). For low income residents such as most PWID, this may have represented a financial burden since those lacking computer or smartphone access were required to pay for computer time and printouts. Additionally, some COVID-19 vaccine queues were very long. People without reliable transportation may have faced additional barriers to access.

An earlier study conducted by our binational team found that only 7.6% of PWID living on either side of the San Diego-Tijuana border reported having had at least one COVID-19 vaccine dose by September 2021, and nearly one-third reported being vaccine hesitant (17). There was no difference in COVID-19 vaccine hesitancy between PWID residing in San Diego vs. Tijuana or by race/ethnicity but younger PWID and those who endorsed COVID-19 disinformation were more vaccine hesitant (17). Disinformation has been described as the deliberate spread of false information, as opposed to misinformation, which is spread without malicious intent (2, 20).

In an effort to increase COVID-19 vaccination among marginalized populations, a temporary pop-up vaccination clinic was set up in Tijuana's Zona Norte that offered assistance to access free COVID-19 vaccination. We studied predictors of COVID-19 vaccine uptake clinic among PWID, hypothesizing that PWID who attended the pop-up clinic would be more likely to be vaccinated. We also postulated that those endorsing more COVID disinformation beliefs would be less likely to become vaccinated.

## Methods

### Participants and eligibility

Between October 28, 2020 and September 10, 2021, adults aged ≥18 or older who injected drugs within the last month and lived in San Diego County or Tijuana were recruited into a longitudinal cohort study, as previously described (2). Recruitment took place using street outreach whereby potential participants were approached in various locations, such as on the street, parks, bars, shelters, motels, river canyons and vacant lots. The current analysis was restricted to the 344 participants who were recruited in Tijuana, had not received a COVID-19 vaccination prior to the opening of the pop-up clinic and who underwent the necessary interviews to collect data on COVID-19 vaccination history. The study was carried out in accordance with The Code of Ethics of the World Medical Association (Declaration of Helsinki). Informed consent was obtained from all participants and the protocol was approved by institutional review board at Xochicalco University in Tijuana.

### Baseline and follow-up survey measures

After providing written informed consent, participants were provided with a photo ID with the study's logo and contact information. All underwent face-to-face interviewer-administered surveys using computer assisted personal interviews in the study office, which was located in the Zona Norte neighborhood in Tijuana. Surveys assessed socio-demographics, chronic health conditions (e.g., diabetes, asthma, hypertension), and potential experiences in their lifetime and during the last 6 months such as homelessness, number of hours spent on the street, injection and non-injection use of specific drugs, food insecurity (21), if they had been enrolled in a substance use treatment program, had been incarcerated or used a syringe services program (SSP).

To reduce participant burden, some survey items, including COVID-19 related beliefs, exposures and vaccination uptake were administered at a supplemental interview approximately 1 week following the baseline visit. We also asked participants about various COVID-19 related experiences (negative income impact, food insecurity, knows someone who died from COVID-19), potential exposures to COVID-19 and protective behaviors (e.g., social distancing, masking, COVID-19 testing). Perceived threat of COVID-19 was assessed by asking participants how worried they were about getting COVID-19 (or getting it again) on a 10 point scale (22).

We asked participants if they had ever received one or more doses of COVID-19 vaccine, and if so, to specify the date and location. To assess COVID-19 misinformation, we presented participants with seven statements about SARS-CoV-2 transmission, severity, immunity, symptoms, treatments and vaccines and asked them to classify each statement as “True”, “False,” or “Unsure” (17). These included the following: (1) COVID-19 cannot be easily spread from one person to another; (2) many thousands of people have not died from COVID-19; (3) most people are immune to COVID-19; (4) you can tell someone has COVID-19 from looking at them; (5) there are treatments that can cure COVID-19; (6) COVID-19 is about as dangerous as having the flu; and (7) COVID-19 vaccines are not safe for pregnant women. We then created a binary variable for each statement indicating whether the participant was misinformed or not, grouping “unsure” responses with responses that clearly indicated having endorsed misinformation.

COVID-19 disinformation was assessed through a six-item scale including conspiracy theory items as previously described (23). These included “COVID-19 was created by the pharmaceutical industry” or “the Chinese government”, “childhood vaccines cause autism” (24), as well as three additional items: “COVID-19 vaccines include a tracking device”, “alter DNA”, and “COVID-19 vaccines offered to ‘people like me' are not as safe”. We dichotomized responses to indicate endorsement of disinformation (“True” and “Unsure”) or not (“False”) and summed them into a total score ranging from 0–6. The mean inter-item correlation value was 0.31, which indicates optimal internal consistency (25). We also assessed COVID-19 vaccine hesitancy as Yes versus No or Unsure.

Follow-up visits were conducted every 6 months where the above measures, including COVID-19 vaccine uptake was re-assessed. Participants were compensated $20 USD for the baseline and follow-up surveys and $10 for the supplemental survey.

### SARS-CoV-2 antibody detection

Blood samples were collected by venipuncture. Sera were batched and tested weekly by Genalyte® (San Diego, CA), using their Maverick™ Multi-Antigen Serology Panel (26) that detects IgG and IgM antibodies to five SARS-CoV-2 antigens.

### SARS-CoV-2 RNA detection

Participants were shown how to self-collect anterior nasal swabs in the presence of study staff. Swabs which were placed in 3 mL of viral transport media for temporary storage, before being shipped for testing at the San Diego Center for AIDS Research laboratory. RT-PCR was conducted using a pooling approach based on the Fluxergy system® (Irvine, CA) to detect SARS-CoV-2 RNA.

### HIV and HCV serology

Rapid HIV and HCV tests were conducted using MedMira's Miriad Rapid HIV/HCV Antibody Test (Halifax, Nova Scottia, CA). Reactive and indeterminate tests underwent a second rapid test with Oraquick® HIV or Oraquick® HCV, respectively (Orasure, Bethlehem, PA) and were confirmed by Western Blot at the UC San Diego Centers for AIDS Research.

### Participant referrals

Following the interview and specimen collection, participants were referred to available resources depending on their responses and stated needs (e.g., treatment for HIV, substance use). From June 21—September 20, 2021, participants who indicated that they had not had a prior COVID-19 vaccine were referred to a pop-up COVID-19 vaccine clinic, which was located nearby the study office in a neighborhood known for its high level of drug use and where sex work is quasi-legal.

### Pop-up clinic procedures

Participants who attended the pop-up clinic were permitted to show their photo ID from our study as proof of identification and were provided with assistance obtaining their CURP if needed by clinic staff, all of whom had extensive experience with substance users. Participants were also invited to undergo a short interviewer-administered survey which included reasons why they had not yet received a COVID-19 vaccine. At the end of the survey they were reimbursed $5 USD and were offered facilitated access to free single-dose Janssen® COVID-19 vaccine by a licensed medical provider with pre- and post-test counseling. Monetary reimbursement was not contingent upon participants' decision to receive the vaccine.

### Statistical analysis

All eligible participants who were not vaccinated prior to establishment of the pop-up clinic (i.e., June 21^st^, 2021) were included in this analysis. Participants were followed up until April 5^th^, 2022 and the outcome (i.e., whether they received a vaccine or not by the end of follow-up period) was assessed.

Characteristics of participants who were and were not COVID-19 vaccinated were summarized by generating frequencies and percentages for binary variables and means and standard deviations for continuous variables. The two groups were compared using Mann-Whitney *U* tests for continuous variables and Chi-square or Fisher's Exact tests for categorical variables. Since our primary objective was to assess whether the exposure to an intervention (visiting the pop-up clinic) was successful at increasing vaccination uptake, we undertook the following analytical approach. First, as suggested by VanderWeele (2019) (27), we selected a series of variables to further examine and determine potential confounders to control for in a multivariable model to estimate the intervention effect on the outcome. Initially, all the variables listed in **Table 2** were selected based on subject-matter knowledge and the assumption that any could played a causal role on the outcome (vaccine uptake), primary exposure (visiting the pop-up clinic), or both.

Next, we regressed each individual variable listed in **Table 2** on the vaccine uptake outcome by conducting univariate Poisson regressions with robust standard error estimations *via* generalized estimating equations (GEE) (28, 29). Whether a participant got vaccinated between June 21^st^, 2021 and April 5^th^, 2022 in conjunction with the natural logarithm of time spent “at risk” facilitated the estimation of the vaccine incidence rate. For those who got vaccinated, time spent “at risk” was calculated as the number of days between the dates when COVID-19 vaccination was first offered to the date of self-reported vaccination, whereas for those unvaccinated it was calculated as the number of days between when COVID-19 vaccination was first offered to the date when the participant was last seen. The estimates from the aforementioned regressions are listed in **Table 2**.

To identify variables that might play a causal role on the exposure, we regressed all of the variables listed in **Table 2** on the exposure variable (i.e., attended the pop-up clinic) by conducting univariate logistic regressions with robust standard error estimation *via* GEE (results not shown).Next, considering each variable's effect size on the outcome or exposure, in conjunction with a level of statistical significance of 0.10 which is in an acceptable range supported in the literature (28), we narrowed down the candidates for inclusion in a multivariable model. Last, we created the final multivariable model by using the “purposeful selection of variables” strategy of Hosmer and Lemeshaw (1999, 2000) (30, 31), where subject matter significance, relationships among the independent variables (e.g., correlations, confounding, and interactions) and statistical significance were taken into consideration. In the final multivariable model, only covariates that maintained a significance level <0.10 were retained. All possible confounding interactions were assessed and ruled out. Multi-collinearity was ruled out based on appropriate values of the largest condition index and VIFs.

All statistical analyses were conducted using SAS, version 9.4.

## Results

### Sample characteristics

A total of 344 cohort participants reported not having received any COVID-19 vaccine before June 21, 2021 and completed questions on COVID-19 vaccination during the study period ending April 5, 2021, and hence were eligible for this analysis. The majority were male (74.4%), Mexican (91.0%) and mean age was 43 years (SD = 9.6).

### COVID-19 vaccine hesitancy and vaccine uptake

Of the 344 participants, 324 (94.2%) completed the supplemental survey which included questions on COVID-19 vaccine hesitancy. Of these, 62 (19.1%) reported that they were not interested in receiving the vaccine, 55 (17.0%) were unsure and 207 (63.9%) reported that they were willing to be vaccinated. Over nearly 10 months of follow-up, 136 (39.5%) reported having received at least one COVID-19 vaccine dose, of whom 113 (83.1%) received vaccine at the pop-up clinic and 23 (16.9%) received it elsewhere. Of 105 participants who received COVID-19 vaccine at the pop-up clinic and had previously answered questions on vaccine hesitancy, 36 (34.3%) had previously expressed being unwilling or unsure about being vaccinated against COVID-19.

### Factors associated with COVID-19 vaccine uptake in univariate regression

Factors associated with receiving at least one dose of COVID-19 vaccine during the 10 month follow-up period are shown in Tables 1, 2. We observed no sociodemographic factors that predicted COVID-19 vaccine uptake. Considering behavioral characteristics, participants who reported engaging in sex work in the last 6 months were marginally more likely to receive COVID-19 vaccination (Table 1). Participants who reported using crack cocaine were less likely to have been vaccinated compared to those who did not report use of crack (2.2% vs. 7.7%, *p* = 0.03). Also, participants who reported increasing their handwashing or use of hand sanitizers were more likely to have been vaccinated compared to those who did not (11.0% vs. 3.8%, *p* = 0.01), but no other protective or health-related factors were predictive of vaccine uptake.

**Table 1:** Characteristics Associated with COVID-19 Vaccination among PWID in Tijuana, Mexico (*n* = 344).

**Baseline characteristics**	**Vaccinated *N* = 136**	**Not vaccinated *N* = 208**	**Total *N* = 344**	***P***
***Socio-demographics***	
Male	98 (72.1%)	158 (76.0%)	256 (74.4%)	0.45
Mean Age [standard deviation (SD)]	43.5 (9.6)	43.3 (9.7)	43.4 (9.6)	0.91
Hispanic/Latinx/Mexican	121 (89.0%)	192 (92.3%)	313 (91.0%)	0.34
Speaks English	132 (97.1%)	195 (93.8%)	327 (95.1%)	0.21
Speaks Spanish	78 (57.4%)	111 (53.4%)	189 (54.9%)	0.51
Born in Mexico	108 (79.4%)	156 (75.0%)	264 (76.7%)	0.36
Primary residence in Tijuana	78 (57.4%)	114 (54.8%)	192 (55.8%)	0.66
Mean years of school completed (SD)	8.4 (3.1)	8.7 (3.4)	8.6 (3.3)	0.38
Married or common law	39 (28.7%)	47 (22.6%)	86 (25.0%)	0.21
Average monthly income <500 USD	94 (69.1%)	143 (68.8%)	237 (68.9%)	1.0
***Impact/Exposures related to COVID-19***	
Homeless^*^	42 (30.9%)	66 (31.7%)	108 (31.4%)	0.91
Incarcerated^*^	8 (5.9%)	14 (6.7%)	22 (6.4%)	0.82
Mean # of people in the same household (SD)^*^	5.8 (11.6)	6.3 (12.5)	6.1 (12.2)	0.62
Engaged in sex work^*^	29 (21.3%)	29 (13.9%)	58 (16.9%)	0.08
Client of sex worker^*^	8 (5.9%)	15 (7.2%)	23 (6.7%)	0.67
Income worse since COVID began^Y^	112 (83.0%)	156 (75.4%)	268 (78.4%)	0.11
Low/very low food security since COVID began	115 (84.6%)	177 (85.1%)	292 (84.9%)	0.88
***Substance use***	
Smokes cigarettes	117 (86.0%)	184 (88.5%)	301 (87.5%)	0.51
Smoked or vaped marijuana^*^	61 (44.9%)	112 (53.8%)	173 (50.3%)	0.12
Smoked/snorted/inhaled/vaped methamphetamine^*^	65 (47.8%)	113 (54.3%)	178 (51.7%)	0.27
Smoked/snorted/inhaled crack or powder cocaine^*^	3 (2.2%)	16 (7.7%)	19 (5.5%)	0.03
Smoked/snorted/inhaled/vaped either heroin or fentanyl^*^	23 (16.9%)	42 (20.2%)	65 (18.9%)	0.48
Injected methamphetamine^*^	52 (38.2%)	82 (39.4%)	134 (39.0%)	0.91
Injected cocaine^*^	6 (4.4%)	14 (6.7%)	20 (5.8%)	0.48
Injected either heroin or fentanyl^*^	125 (91.9%)	193 (92.8%)	318 (92.4%)	0.84
Mean # of years of injection drug use (SD)	21.0 (11.5)	21.0 (11.5)	21.0 (11.5)	0.93
Mean # of times injected drugs per day^*^(SD)	2.4 (1.6)	2.6 (1.6)	2.5 (1.6)	0.29
Visited shooting galleries^*^	46 (33.8%)	64 (30.8%)	110 (32.0%)	0.56
Receptive needle sharing^*^	71 (52.2%)	121 (58.2%)	192 (55.8%)	0.32
***Health conditions***	
Tested HIV+	19 (14.0%)	27 (13.0%)	46 (13.4%)	0.87
Tested HCV+	55 (40.4%)	82 (39.4%)	137 (39.8%)	0.91
Mean # of chronic conditions (excluding seasonal allergies and acne/skin problems) (SD)	0.5 (0.9)	0.7 (1.2)	0.6 (1.1)	0.25
Has at least one chronic illness	39 (28.7%)	69 (33.2%)	108 (31.4%)	0.41
***Protective behaviors during the COVID-19 pandemic***	
Social Distancing	9 (6.6%)	7 (3.4%)	16 (4.7%)	0.19
Isolated or quarantined themselves	2 (1.5%)	3 (1.4%)	5 (1.5%)	1.0
Wore face mask	111 (81.6%)	157 (75.5%)	268 (77.9%)	0.19
Increased handwashing/sanitizer	15 (11.0%)	8 (3.8%)	23 (6.7%)	0.01
Engaged in at least one protective behavior	119 (87.5%)	178 (85.6%)	297 (86.3%)	0.63
***COVID-19-related disinformation***	
Thinks that the pharmaceutical industry created the COVID-19 virus	36 (26.5%)	49 (23.6%)	85 (24.7%)	0.61
Thinks that the coronavirus was created by the Chinese government as a biological weapon	43 (31.6%)	63 (30.3%)	106 (30.8%)	0.81
Thinks that vaccines given to children for diseases like measles and mumps cause autism	90 (66.2%)	112 (53.8%)	202 (58.7%)	0.03
Thinks that COVID-19 vaccines being offered to 'people like me' are not as safe as other COVID-19 vaccines	17 (12.5%)	27 (13.0%)	44 (12.8%)	1.0
Thinks that COVID-19 vaccines include a tracking device	16 (11.8%)	33 (15.9%)	49 (14.2%)	0.34
Thinks that some COVID vaccines could change their DNA	18 (13.2%)	25 (12.0%)	43 (12.5%)	0.74
Endorses at least one conspiracy theory	101 (74.3%)	128 (61.5%)	229 (66.6%)	0.01
***COVID-19-related misinformation***	
Does not think that the virus that causes COVID-19 can be easily spread from one person to another	29 (21.3%)	44 (21.2%)	73 (21.2%)	1.0
Does not think that many thousands of people have died from COVID-19	25 (18.4%)	34 (16.3%)	59 (17.2%)	0.66
Thinks that most people already have immunity to COVID-19	98 (72.1%)	133 (63.9%)	231 (67.2%)	0.13
Thinks that you can tell someone has COVID-19 by looking at them	24 (17.6%)	30 (14.4%)	54 (15.7%)	0.45
Thinks that having COVID-19 is about as dangerous as having the flu	47 (34.6%)	70 (33.7%)	117 (34.0%)	0.91
Does not think that COVID-19 vaccines are safe for pregnant women	54 (39.7%)	91 (43.8%)	145 (42.2%)	0.50
***Most important source of COVID-19-related*** ***Information***	
Friends	80 (58.8%)	129 (62.0%)	209 (60.8%)	0.57
Doctors/health professionals	3 (2.2%)	1 (0.5%)	4 (1.2%)	0.30
Social media	11 (8.1%)	20 (9.6%)	31 (9.0%)	0.70
Conservative TV/radio	38 (27.9%)	52 (25.0%)	90 (26.2%)	0.62
Liberal TV/radio	4 (2.9%)	6 (2.9%)	10 (2.9%)	1.0
***Additional COVID-19-related experiences***	
Visited pop-up COVID-19 vaccine clinic	117 (86.0%)	63 (30.3%)	180 (52.3%)	< .001
COVID-19 Vaccine hesitancy (yes vs. no/unsure)^Y2^	32 (24.1%)	64 (30.8%)	96 (28.2%)	0.22
Knows someone who died from COVID-19	27 (19.9%)	39 (18.8%)	66 (19.2%)	0.89
Mean for: On a scale of 1 (low) to 10 (very), how worried are you of getting COVID-19 or getting it again (SD)	5.7 (2.8)	5.7 (2.6)	5.7 (2.7)	0.89
Knows someone who was vaccinated for COVID-19	93 (68.4%)	113 (54.3%)	206 (59.9%)	0.01
Thinks they had COVID-19	5 (3.7%)	21 (10.1%)	26 (7.6%)	0.04
Has been tested for COVID-19 outside the study	24 (17.6%)	30 (14.4%)	54 (15.7%)	0.45
Was exposed to somebody who tested positive for COVID-19	7 (5.1%)	12 (5.8%)	19 (5.5%)	1.0
Had at least one COVID-19 symptom on day of interview	37 (27.2%)	58 (27.9%)	95 (27.6%)	0.90
Tested SARS-CoV-2 seropositive	33 (24.3%)	60 (28.8%)	93 (27.0%)	0.09
Tested SARS-CoV-2 RNA positive	0 (0%)	3 (1.4%)	3 (0.9%)	0.28
Ever had a flu vaccine	31 (22.8%)	45 (21.6%)	76 (22.1%)	0.79

* Past 6 months; Missing values, ^Y^n = 2 ^Y2^n = 20.

Between group differences were determined using Mann-Whitney U tests for continuous variables and Chi-square or Fisher's Exact tests for categorical variables.

**Table 2:** Factors Associated with COVID-19 Vaccination among PWID in Tijuana, Mexico.

**Baseline characteristics**	**Univariate RR (95% CI)**	Multivariate Adj RR (95%CI)^**^
***Socio-demographics***	
Male	0.89 (0.67–1.18)	
Age^¥^	1.00 (0.99–1.02)	
Hispanic/Latinx/Mexican	0.80 (0.54–1.18)	
Speaks English	1.72 (0.72–4.08)	
Speaks Spanish	1.10 (0.85–1.44)	
Born in Mexico	1.17 (0.84–1.63)	
Highest year of school completed^¥^	0.98 (0.95–1.02)	
Married or common law	1.21 (0.91–1.60)	
Monthly income <500 USD	1.01 (0.76–1.34)	
***Potential COVID-19 exposures***	
Homeless^*^	0.98 (0.74–1.30)	
Incarcerated^*^	0.91 (0.52–1.62)	
Engaged in sex work^*^^P^	1.34 (0.99–1.80)	1.61 (1.01–2.55)^c^
Client of sex worker^*^	0.87 (0.49–1.55)	
Income worse since COVID began^Y2^	1.34 (0.93–1.94)	
Low/very low food security since COVID began	0.98 (0.68–1.40)	
***Substance use***	
Smokes cigarettes	0.88 (0.61–1.27)	
Smoked or vaped marijuana^*^	0.80 (0.62–1.05)	
Smoked/snorted/inhaled/vaped methamphetamine^*^	0.85 (0.66–1.11)	
Smoked/snorted/inhaled crack or powder cocaine^*^^P^	0.39 (0.14–1.10)	
Smoked/snorted/inhaled/vaped either heroin or fentanyl^*^	0.87 (0.61–1.25)	
Injected methamphetamine^*^	0.97 (0.74–1.27)	
Injected cocaine^*^	0.75 (0.38–1.48)	
Injected either heroin or fentanyl^*^	0.93 (0.58–1.49)	
# of times injected drugs per day^¥^^*^	0.96 (0.89–1.04)	
***Health conditions***	
Tested HIV+	1.05 (0.72–1.53)	
Tested HCV+	1.03 (0.79–1.34)	
Has at least one chronic illness	0.88 (0.65–1.18)	
***Protective behaviors during the COVID-19 pandemic***	
Social Distancing	1.45 (0.92–2.29)	
Isolated or quarantined themselves	1.01 (0.34–2.98)	
Wore face mask	1.26 (0.89–1.79)	
Increased handwashing/sanitizer^P^	1.73 (1.24–2.41)	
Engaged in at least one protective behavior	1.11 (0.74–1.66)	
***COVID-19-related disinformation (i.e., endorsement of*** ***conspiracy theories)***	
Thinks that the pharmaceutical industry created the COVID-19 virus	1.10 (0.82–1.47)	
Thinks that the coronavirus was created by the Chinese government as a biological weapon	1.04 (0.78–1.37)	
Thinks that vaccines given to children for diseases like measles and mumps cause autism	1.38 (1.04–1.83)	
Thinks that COVID-19 vaccines being offered to 'people like me' are not as safe as other COVID-19 vaccines	0.97 (0.65–1.45)	
Thinks that COVID-19 vaccines include a tracking device	0.80 (0.52–1.23)	
Thinks that some COVID vaccines could change their DNA	1.07 (0.73–1.56)	
# of conspiracies they believe (out of 6)^¥^	1.03 (0.96–1.10)	
***COVID-19-related misinformation (i.e., incorrect*** ***knowledge items)***	
Does not think that the virus that causes COVID-19 can be easily spread from one person to another	1.01 (0.73–1.38)	
Does not think that many thousands of people have died from COVID-19	1.09 (0.78–1.52)	
Thinks that most people already have immunity to COVID-19	1.26 (0.94–1.70)	
Thinks that you can tell someone has COVID-19 by looking at them	1.15 (0.83–1.60)	
Thinks that having COVID-19 is about as dangerous as having the flu	1.02 (0.78–1.35)	
Does not think that COVID-19 vaccines are safe for pregnant women	0.90 (0.69–1.18)	
***Most important source of COVID-19-related*** ***Information***	
Friends	0.92 (0.71–1.20)	
Doctors/health professionals^P^	1.92 (1.07–3.43)	1.92 (1.28–2.86)^c^
Social media	0.89 (0.54–1.46)	
Conservative TV/radio	1.09 (0.82–1.46)	
Liberal TV/radio	1.01 (0.47–2.19)	
***Additional COVID-19-related experiences***	
Visited pop-up COVID-19 vaccine clinic^P^	5.61 (3.63–8.68)	9.15 (5.68–14.74)
COVID-19 Vaccine hesitancy^Y2^	0.81 (0.59–1.11)	
Knows someone who died from COVID-19	1.04 (0.75–1.44)	
On a scale of 1 (low) to 10 (very), how worried are you of getting COVID-19 or getting it again^¥^	1.00 (0.95–1.05)	
Knows someone who was vaccinated for COVID-19^P^	1.45 (1.08–1.94)	1.45 (0.97–2.18)^c^
Thinks they had COVID-19^P^	0.47 (0.21–1.04)	0.36 (0.13–1.01)^c^
Has been tested for COVID-19 outside of our study	1.15 (0.83–1.60)	
Has been exposed to somebody with a positive COVID-19 test result	0.93 (0.51–1.70)	
Had at least one COVID-19 symptom on day of interview	0.98 (0.73–1.32)	
Tested SARS-CoV-2 seropositive	0.82 (0.6–1.13)	
Tested SARS-CoV- RNA positive^***^	N/A	
Ever had a flu vaccine	1.04 (0.76–1.42)	

* past 6 months; Missing values, ^Y^n = 2; ^Y2^n = 20; ^¥^Per one unit increase; ^P^P-value <0.10;

** corresponding estimates were adjusted for all the variables included in the multivariable model.

*** N/A due to zero cell for vaccinated persons. ^c^Should be interpreted with caution as this variable was included as a confounder and may not be associated with the outcome.

Surprisingly, participants who received COVID-19 vaccination were more likely to believe that childhood vaccines caused autism (66.2% vs. 53.8%, *p* = 0.03); however, no other COVID-19 disinformation measures were associated with COVID-19 vaccination. Similarly, we found no associations between COVID-19 vaccination and endorsing statements that reflect COVID-19 misinformation. COVID-19 vaccine hesitancy was not significantly associated with vaccine uptake.

The strongest predictor of COVID-19 vaccination was visiting the pop-up COVID-19 vaccine clinic after receiving a referral. Of those who received the COVID-19 vaccine, 86% visited the pop-up clinic, whereas only 30.3% of those unvaccinated visited the clinic (*p* <0.001), which corresponded to a Rate Ratio [RR] of 5.61 95% Confidence Interval (CI): 3.63–8.68; Table 2.

Knowing someone who had received COVID-19 was significantly associated with getting vaccinated, (RR: 1.45; 95% CI: 1.08–1.94) but participants who believed they had already had COVID-19 were less likely to get vaccinated (RR: 0.47; 95%CI: 0.21–1.04). Testing positive for SARS-CoV-2 antibodies or RNA was not associated with vaccination. Association between Pop-Up Clinic Attendance on COVID-19 Vaccine Uptake 3.5.Adjusting for Confounders

Of variables included in Table 2, obtaining most of their COVID−19 information from health providers, having had COVID-19, knowing more people who had received COVID-19 vaccines and engaging in sex work were identified as potential confounders. After adjusting for these variables, attending the pop-up clinic became even more strongly associated with COVID-19 vaccination in the multivariate model [Adjusted Rate Ratio (AdjRR: 9.15; 95% CI: 5.68–14.74), Adjustment for sociodemographic factors such as age and sex did not alter parameter estimates. No significant interactions were observed.

## Discussion

In this prospective study of PWID living in Tijuana during the COVID-19 epidemic, we found that attending a temporary pop-up vaccine clinic was independently associated with greater uptake of COVID-19 vaccination. Before this clinic was established, <10% of PWID in our study had received at least one COVID-19 vaccine dose (17), which is similar to a study of PWID in Oregon, USA (32). By the end of the 10 months follow-up period, the proportion of participants who had received at least one COVID-19 vaccine dose had increased to 39.5%. Although this level of vaccine coverage remains grossly sub-optimal, it is encouraging that at least one third of the study participants who were vaccinated during the pop-up clinic had previously reported being vaccine hesitant, suggesting that attendance at the clinic was influential in their decision to receive COIVID-19 vaccine.

Our findings should be interpreted cautiously due to the observational nature of our cohort study. Due to time constraints and the public health imperative to increase COVID-19 vaccination in this highly vulnerable population, we did not randomize participants to receive pop-up clinic referrals in a clinical trial design, which would have been more rigorous. Our study may have under-estimated the impact of the pop-up COVID-19 vaccination clinic since the clinic only operated during the first two months of the follow-up period and low statistical power may have attenuated the magnitude of some associations. Also, while we created a single multivariable model to assess the effect of the intervention on the outcome and adjusted for potential confounders which is common practice, some authors (33) suggest caution when trying to interpret the effects of the confounders on the outcome as they may not have the same interpretation as that of a “primary effect” on the outcome. We therefore limit our discussion to the potential effect of the pop-up clinic on COVID-19 vaccine uptake.

It is possible that some participants accepted the referral to the pop-up clinic because they were more interested in the $5 monetary reimbursement for completing the supplemental survey, rather than receiving COVID-19 vaccine. Indeed, a review of 11 clinical studies showed that financial incentives were associated with a seven-fold increase in adherence to the vaccine schedule for Hepatitis B virus, leading some researchers to advocate for contingency management to increase COVID-19 vaccine uptake (8, 34). In a study of PWID in Oregon, contingency management was associated with a significant increase in SARS-CoV-2 testing (35). However, financial incentives are not able to compensate for broad vaccination barriers (36), and some studies have shown that small compensations may not increase COVID-19 vaccination rates (37, 38). Therefore, it may not be realistic to expect that lower and middle-income countries could provide large enough financial incentives to significantly increase COVID-19 vaccination given limited resources and competing health priorities.

Despite the limitations of our study design and relatively short follow-up period, our findings have implications for improved COVID-19 vaccine uptake in this marginalized population. Greater uptake of COVID-19 vaccination associated with the pop-up clinic could have been due to its convenient location in the Zona Norte, the clinic staff's experience and familiarity with the issues facing people living with addiction, the ability of participants to use their photo ID from the study as proof of identification, or the assistance some participants received from clinic staff in obtaining their CURP. Since our observational study design was unable to determine which of these or other factors may have been most influential in the participants' decisions to receive vaccination, additional studies are required to examine client preferences to appropriately tailor services to their needs.

Although we did not find COVID-19 disinformation, misinformation or vaccine hesitancy to be significantly associated with lower vaccine uptake as we had hypothesized, it is noteworthy that two-thirds of our cohort endorsed at least one COVID-19 conspiracy theory, one third felt that COVID-19 was “no worse than the flu”, and close to 50% believed that COVID-19 vaccines are not safe for pregnant women. Apart from system-level barriers, widespread COVID-19 disinformation and government criticism was prevalent on both sides of the US-Mexico border (39) making it harder for people to discern false information from evidence-based sources. Based on these findings, additional interventions to address medical mistrust are needed.

In summary, our prospective evaluation found a significant increase in COVID-19 vaccine uptake associated with attending a pop-up vaccine clinic in Tijuana. Despite our encouraging findings, <50% of PWID in our study had received at least one COVID-19 vaccine dose. Sustained efforts to improve COVID-19 vaccination in this population should focus on removing logistical and structural barriers to ensure that their health and that of the general population are protected.

## Data availability statement

The datasets presented in this article are not readily available because the study is ongoing. Requests to access the datasets should be directed to DA, dabramovitz@health.ucsd.edu.

## Ethics statement

The studies involving human participants were reviewed and approved by Institutional Review Board at Xochicalco University and the Office of IRB Administration at the University of California San Diego. The patients/participants provided their written informed consent to participate in this study.

## Author contributions

AH-V, SM, MR, and CR were responsible for participant referrals and data collection. DA and IA were responsible for data management and analysis. SS conceived the study design and oversaw the analysis. SS, AH-V, and DA interpreted the findings. MM, SS, AH-V, IA, and SM wrote the manuscript. AH-V, SM, IA, DA, MM, CR, SA, and MR reviewed and edited the manuscript. All authors contributed to the article and approved the submitted version.

## Funding

This work was supported by the National Institute on Drug Abuse (NIDA) at the National Institutes of Health (NIH) (R01DA049644-S1). Additional support was provided by the National Institute of Allergy and Infectious Diseases (P30 AI036214). UC San Diego Altman Clinical and Translational Research Institute SUSTAIN program (NIH/NCATS 1KL2TR001444).

## Conflict of interest

The authors declare that the research was conducted in the absence of any commercial or financial relationships that could be construed as a potential conflict of interest.

## Publisher's note

All claims expressed in this article are solely those of the authors and do not necessarily represent those of their affiliated organizations, or those of the publisher, the editors and the reviewers. Any product that may be evaluated in this article, or claim that may be made by its manufacturer, is not guaranteed or endorsed by the publisher.
